# Abstract from Professor Hunting’s Remarks on Pelvic Fractures in the Horse

**Published:** 1901-11

**Authors:** 


### ABSTRACT FROM PROFESSOR HUNTING'S REMARKS ON PELVIC FRACTURES IN THE HORSE.

MR. HUNTING commenced his short address by reference to the anatomy of the pelvis—a bony ring which supported the weight of the posterior half of the body. This ring had processes jutting out from it, and fractures of these were not, as a rule, serious. The grave fractures were those which broke the continuity of the ring, and were consequently greatly displaced. He next discussed the cause of pelvic fractures, and concluded that nearly all arose as the direct result of falls or external violence, but some were due to muscular action. This appeared perhaps more often when the parts were weakened by a previous fracture. For instance, in cases of fracture of the pubis into the obturator foramen, lameness might be slight, and yet a horse jumping, suddenly stopping, or twisting around, as in polo, might directly fracture the shaft of the ilium.

This led to remarks on the varieties of fracture, especially multiple fractures, and on the accidents which accompanied or resulted from fracture, such as division of the iliac bloodvessels, to injury of the obturator nerve, and to perforations of the bladder, rectum, or vagina. Then the different fractures were taken individually and any special point referred to. In order of their frequency, they were of the external angle of the ilium, shaft of the ilium, bridge of the pubis, tuberosity of the ischium, the symphysis, and, very rarely, the internal angle of the ilium, through the ischium into the obturator foramen.

Mr. Hunting devoted some time to the question of diagnosis, and said that although we might probably always, by care, diagnose a fracture of the pelvis, we could not always locate the exact position of the lesion. Very difficult were the cases which were down and could not rise. In street accidents there was great risk of error if a hasty diagnosis were given. When the horse stood on one hind leg it was easier to make a proper examination. Crepitation was, of course, most diagnostic, but sometimes it was absent in cases of fracture of the pelvis. Quivering of the muscles over the fracture was, he thought, a most indicative symptom. Deformity when present was helpful, but it was seldom marked except when great displacement occurred. Examination per rectum should never be neglected. In mares when the symphysis or floor of the pelvis was suspected the examination should be per vaginam.

In most pelvic fractures some effusion took place at the seat of injury. In those affecting the floor of the pelvis this effusion was marked by swelling in the perineal regoin or in front of the sheath or udder, as the fracture was posteriorly or anteriorly situated. This swelling he considered very diagnostic.—*Veterinary Record*, November 9, 1901.

